# A mixed methods exploration of the interrelationships among self-compassion, stress management, psychological capital, and life satisfaction in Chinese university students

**DOI:** 10.3389/fpsyg.2025.1510987

**Published:** 2025-06-03

**Authors:** Ping Huang, Zhenxing Lin, BingRu Wang, Zhou Du

**Affiliations:** ^1^Graduate School, Wenzhou Medical University, Wenzhou, Zhejiang, China; ^2^Department of Student Affairs, Wenzhou University of Technology, Wenzhou, Zhejiang, China; ^3^The First Affiliated Hospital of Wenzhou Medical University, Wenzhou, Zhejiang, China

**Keywords:** self-compassion, stress management, psychological capital, life satisfaction, mixed-methods, structural equation modeling, emotional resilience

## Abstract

**Introduction:**

This study explored the interrelationships between self-compassion, stress management, psychological capital (PsyCap), and life satisfaction among Chinese university students.

**Methods:**

A mixed-methods approach was employed. The quantitative phase involved 478 students from six universities, with data analyzed using Structural Equation Modeling (SEM) to test hypothesized direct and indirect relationships among the variables. The qualitative phase included semi-structured interviews with a subsample of 30 participants.

**Results:**

SEM results indicated that self-compassion and stress management positively influenced life satisfaction, both directly and indirectly, with PsyCap (comprising resilience, optimism, and self-efficacy) acting as a key mediator. PsyCap was strongly associated with higher life satisfaction. Multi-group SEM analyses showed no significant gender differences in the relationships among the variables. Qualitative findings offered deeper insights into students’ experiences, highlighting challenges in balancing self-compassion with cultural and academic pressures, and confirmed PsyCap’s role as a protective factor.

**Discussion:**

The findings emphasize the importance of promoting self-compassion, adaptive stress management strategies, and psychological capital to enhance the well-being of university students, particularly in high-stress academic environments.

## Introduction

University students face significant academic stress, which impacts their mental health and life satisfaction (Cabras and Mondo, 2018; Luthans et al., 2007; Prasath et al., 2022; Seppälä et al., 2020). This stress, often linked to anxiety, depression, and burnout (Ciarrochi et al., 2002; Rabenu et al., 2017), is heightened in collectivist cultures like China, where academic success ties to family honor and societal expectations (Cheung et al., 2016; López et al., 2018). Understanding psychological resources that help students manage these pressures is vital.

Self-compassion, involving kindness toward oneself during failure and recognizing shared human experiences, supports emotional regulation and resilience (Germer and Neff, 2013; Neff, 2003). It reduces self-criticism and boosts life satisfaction through adaptive coping strategies like mindfulness and positive reappraisal (Homan, 2016; Neff and Vonk, 2009; Richardson and Rothstein, 2008). Recent research has increasingly explored self-compassion in Chinese populations (e.g., Li et al., 2021; Wang and Lou, 2022; Chen, 2021), highlighting its role in wellbeing across diverse groups, such as self-quarantined residents during the COVID-19 pandemic and overseas Chinese students. Still, its interplay with resources like PsyCap and stress management in Chinese university students remains unclear.

Psychological Capital (PsyCap), encompassing self-efficacy, hope, resilience, and optimism, enhances wellbeing by aiding stress coping and goal pursuit (Avey et al., 2010; Culbertson et al., 2010; Luthans et al., 2007). Higher PsyCap levels correlate with adaptive coping strategies, such as problem-solving and mindfulness, improving stress management and life satisfaction (De Vibe et al., 2013; Flinchbaugh et al., 2015). While PsyCap has been studied in Chinese academic settings (e.g., Cao et al., 2024; Wang et al., 2023; Sun et al., 2022), particularly among PhD students and nursing students, its mediating role among self-compassion, stress management, and life satisfaction is underexplored.

Stress management, through adaptive strategies like mindfulness and social support, is critical for students’ wellbeing (Querstret et al., 2020; Smith and Yang, 2017), while maladaptive strategies like avoidance increase distress (Ciarrochi et al., 2002; Roohafza et al., 2016). It is especially relevant in university settings, where students balance academic and personal demands (Freire et al., 2016; Roohafza et al., 2016). Recent studies on Chinese students (e.g., Jing et al., 2022; Shan et al., 2022) have emphasized stress management and coping styles, yet their interaction with self-compassion and PsyCap remains insufficiently investigated.

Despite progress, gaps persist. Research often examines self-compassion, PsyCap, and stress management separately or in specific groups (e.g., Cao et al., 2024; Li et al., 2021; Wang et al., 2023), neglecting their interrelationships in a broader sample of Chinese university students. Mixed-methods studies combining quantitative and qualitative data are scarce. While direct links between self-compassion, PsyCap, and life satisfaction are known, PsyCap’s mediating role is unclear. Additionally, reconciling self-compassion with cultural norms favoring self-criticism for success in China remains unaddressed (Homan, 2016; López et al., 2018). These gaps highlight the need for culturally sensitive interventions.

This study investigates the relationships among self-compassion, stress management, PsyCap, and life satisfaction in Chinese university students, focusing on PsyCap’s mediating role. Using a mixed-methods approach, it combines quantitative and qualitative data to explore how cultural norms shape these constructs and students’ coping strategies.

## Literature review

### Self-compassion and emotional resilience

Self-compassion, rooted in Buddhist traditions, involves treating oneself with kindness during failure or inadequacy rather than engaging in harsh self-criticism (Allen and Leary, 2010; Germer and Neff, 2013; Neff, 2003). Neff’s (2003) model includes self-kindness, common humanity, and mindfulness, which together build emotional resilience by helping individuals see suffering as a shared experience and respond to distress with mindful awareness (Neff, 2023; Neff and Knox, 2020). This compassionate approach not only mitigates immediate emotional distress but also establishes a foundation for enduring psychological strength.

Research shows self-compassion aids emotional regulation, reducing rumination and self-blame (Diedrich et al., 2014; Neff, 2011; Zessin et al., 2015). By decreasing negative self-focused thoughts, it lessens distress and fosters adaptive responses to stress (Marshall and Brockman, 2016; Terry and Leary, 2011), thereby enhancing psychological stability and enabling individuals to confront challenges more effectively (Germer and Neff, 2013; Marsh et al., 2018). These emotional regulation benefits extend to psychological capital—a set of resources including resilience, optimism, and wellbeing—which collectively improve stress management and life satisfaction (Avey et al., 2009; Finlay-Jones et al., 2015; Luthans et al., 2007; Rabenu et al., 2017).

Self-compassion bolsters emotional regulation, a key part of wellbeing (Crego et al., 2022; Inwood and Ferrari, 2018; Zhang and Fathi, 2024), by promoting mindful awareness that prevents maladaptive coping like avoidance (Bakker et al., 2019; Finlay-Jones et al., 2015; Kramer et al., 2023). This lowers anxiety and depression (Ewert et al., 2021; Zessin et al., 2015) and builds resilience and optimism, which are essential for life satisfaction. Self-compassion also boosts life satisfaction directly by reducing self-criticism and framing imperfections as universal (Hall et al., 2013; Mantelou and Karakasidou, 2017; Neff, 2003; Yang et al., 2016). It cushions daily stressors, enhancing self-concept and wellbeing (López et al., 2018; Pandey et al., 2021; Zessin et al., 2015), with interventions like mindfulness training showing long-term gains (Tran et al., 2024; Watson, 2024).

Cross-cultural studies confirm these benefits. Among Malaysian counselors, self-compassion increased resilience, supporting life satisfaction (Voon et al., 2022), showing its value across diverse settings (Bluth et al., 2017; Kotera and Van Gordon, 2021). In summary, self-compassion improves emotional regulation, stress management, and life satisfaction by fostering resilience and psychological capital. Its broad relevance makes it a critical focus for wellbeing interventions, especially in high-stress environments.

### The role of stress management in mental health

Stress management involves strategies to regulate the emotional, psychological, and physical effects of stressors (Barlow, 2007; Seaward, 2017; Wright et al., 2013). According to Lazarus and Folkman’s (1984) transactional model, stress arises when individuals perceive an imbalance between situational demands and their coping abilities. This model is particularly relevant in academic settings, where students often face stress from performance and social pressures. Coping strategies—conscious efforts to handle stress—are categorized as adaptive or maladaptive. Adaptive strategies, like problem-solving and positive reappraisal, foster constructive engagement with stressors, enhancing wellbeing (Richardson and Rothstein, 2008). Maladaptive strategies, such as avoidance, may worsen stress and lead to emotional distress (Ciarrochi et al., 2002; Roohafza et al., 2016).

Adaptive coping addresses stress sources directly (Zeidner, 1995), using techniques like problem-solving, social support, and cognitive reappraisal. Problem-solving reduces stress by identifying solutions (Lazarus and Folkman, 1984; Murphy, 1996), while mindfulness-based practices, such as Mindfulness-Based Stress Reduction (MBSR), promote emotional regulation through present-moment awareness (De Vibe et al., 2013; Querstret et al., 2020). These strategies enhance wellbeing and life satisfaction by fostering control over stressors (De Witte et al., 2019; Morales-Rodríguez et al., 2020) and building psychological capital—resilience and optimism—central to life satisfaction (De Vibe et al., 2013; Ewert et al., 2021; Rabenu et al., 2017; Richardson and Rothstein, 2008).

Conversely, maladaptive strategies like avoidance or denial offer short-term relief but often exacerbate long-term psychological outcomes (Bonne et al., 2004; Sirois and Kitner, 2015). Avoidance, for example, prevents addressing stress causes, leading to prolonged distress and burnout (Suleman et al., 2018; Roohafza et al., 2016). Reliance on such strategies is linked to emotional exhaustion, especially in high-stress environments (Bittner et al., 2011; Richardson and Rothstein, 2008; Simpson et al., 2019). A meta-analysis by Richardson and Rothstein (2008) found that maladaptive coping predicts higher mental health disorder rates in chronically stressful settings.

Effective stress management is crucial for mental health and life satisfaction, a key aspect of subjective wellbeing (Madazimova and Mambetalina, 2024). Adaptive coping equips individuals to handle stress, reducing psychological distress and improving life satisfaction (De Vibe et al., 2013; De Witte et al., 2019; Morales-Rodríguez et al., 2020; Roohafza et al., 2016). Mindfulness interventions, for instance, lower anxiety and depression by enhancing emotional regulation (Querstret et al., 2020). In contrast, maladaptive coping correlates with lower life satisfaction, as it avoids addressing stress’s root causes (Ciarrochi et al., 2002; Faul et al., 2010; Wicks, 2005; Suleman et al., 2018). Thus, promoting adaptive strategies is vital for mental health and life satisfaction, particularly among university students facing academic and social stressors. This aligns with the study’s focus on how stress management interacts with self-compassion, psychological capital, and life satisfaction to support students in high-pressure environments (Riepenhausen et al., 2022).

### Psychological capital and wellbeing

Psychological Capital (PsyCap) refers to an individual’s positive psychological state, consisting of four core, interrelated dimensions: self-efficacy, hope, resilience, and optimism (Loghman et al., 2023; Luthans et al., 2007; Newman et al., 2014). These dimensions function together, strengthening one another to provide the mental resources needed to handle challenges, improve wellbeing, and support success in various contexts (Avey et al., 2009). In university settings, where students face academic pressure and personal stress, PsyCap plays a key role in helping them cope, stay motivated, and maintain emotional health (Bandura, 1997; Luthans and Youssef-Morgan, 2017).

Each dimension of psychological capital contributes distinctly to psychological health, working together to support students in unique ways. Self-efficacy, the confidence in one’s ability to succeed, drives persistence and reduces stress by giving students a sense of control over tasks like exams or assignments, as Bandura (1997) emphasizes. This control seamlessly connects to hope, which involves the will to achieve goals and the ability to find ways to reach them, helping students stay focused and adapt to setbacks in high-pressure academic environments, according to Snyder (2002). As students navigate these challenges, resilience steps in, enabling recovery from difficulties—such as a failed test—allowing them to bounce back and maintain emotional balance, a process Masten (2001) highlights as critical. Meanwhile, optimism, the belief in positive outcomes, encourages a forward-looking attitude that sustains motivation and wellbeing even during tough times, as Scheier and Carver (1985) describe, rounding out PsyCap’s comprehensive support for psychological health.

Research shows PsyCap lowers stress, anxiety, and depression while boosting life satisfaction (Culbertson et al., 2010; Rabenu et al., 2017). Self-efficacy counters helplessness, optimism keeps spirits up, hope pushes goal pursuit, and resilience protects against burnout (Fathi et al., 2023), creating a combined effect that enhances mental health and a sense of fulfillment. Studies highlight these benefits in various groups, such as student-athletes managing dual demands (Kim et al., 2020), medical students facing intense training (Nafees and Jahan, 2017), and leaders in stressful roles (Gyu Park et al., 2017; Roche et al., 2014). For Chinese university students, who often deal with high academic expectations and cultural demands, PsyCap may be especially important for sustaining wellbeing and life satisfaction.

The value of PsyCap is clear in high-stress settings like universities. It reduces psychological distress in international students adjusting to new surroundings (Prasath et al., 2022) and links spiritual wellbeing to mental health in nursing students (Parviniannasab et al., 2022). Because PsyCap can be developed, interventions like resilience workshops or cognitive techniques to reframe negative thoughts offer practical ways to build these strengths, improving students’ ability to cope and thrive (Amini et al., 2020).

In summary, PsyCap is vital for resilience, wellbeing, and life satisfaction. Its dimensions—self-efficacy, hope, resilience, and optimism—work together to help individuals manage stress and succeed in demanding environments. Strengthening PsyCap through targeted efforts could greatly benefit university students, particularly in high-stress academic contexts, enhancing their psychological health and overall satisfaction.

### Life satisfaction and psychological resources

Life satisfaction, a central component of subjective wellbeing, reflects an individual’s cognitive appraisal of their overall quality of life (Diener et al., 1985). Unlike transient emotional states, it offers a stable, long-term evaluation (Ehrhardt et al., 2000), integrating both hedonic (pleasure-oriented) and eudaimonic (meaning-driven) aspects of wellbeing (Martela and Sheldon, 2019; Ryan and Deci, 2001; Zhang et al., 2023). Key psychological resources—self-compassion, PsyCap, and effective stress management—play a significant role in shaping life satisfaction by fostering resilience, adaptive coping, and emotional stability (Avey et al., 2009; Homan, 2016).

Self-compassion, which involves treating oneself with kindness during setbacks, strengthens emotional resilience, reduces self-criticism, and enhances overall wellbeing, thereby contributing to higher life satisfaction (Neff, 2011; Cho et al., 2021). Studies demonstrate a positive association between self-compassion and life satisfaction across diverse age groups (Yang et al., 2016; Kim and Ko, 2018), suggesting that self-compassion supports emotional stability and facilitates effective coping with stress, both of which are essential for a fulfilling life.

PsyCap, a multifaceted construct encompassing self-efficacy, hope, resilience, and optimism, further promotes life satisfaction by equipping individuals with the tools to manage stress, pursue meaningful goals, and maintain a positive outlook (Datu and Valdez, 2016; Hite, 2015). For instance, during the COVID-19 pandemic, self-compassion and positive coping—integral elements of PsyCap—were associated with higher life satisfaction (Li et al., 2021). Specifically, hope, a core component of PsyCap, drives goal attainment and acts as a buffer against emotional challenges, thereby enhancing life satisfaction (Cotton Bronk et al., 2009; Gungor and Avci, 2017; Mantelou and Karakasidou, 2017).

Complementing these resources, effective stress management through adaptive strategies such as problem-solving and seeking social support is crucial for sustaining wellbeing and life satisfaction (Flinchbaugh et al., 2015; Smith and Yang, 2017). Interventions like mindfulness and cognitive-behavioral approaches have been shown to enhance emotional regulation and resilience, leading to increased life satisfaction (Clabaugh et al., 2021; Uraz et al., 2013; Freire et al., 2016). In contrast, reliance on maladaptive coping strategies, such as avoidance, is associated with reduced life satisfaction (Querstret et al., 2020).

In conclusion, self-compassion, PsyCap, and adaptive stress management are essential for fostering life satisfaction by helping individuals navigate challenges and pursue meaningful goals. The interplay of these resources creates a robust framework for emotional and psychological resilience, making interventions that target them particularly valuable in high-stress environments like universities, which aligns with the focus of this study.

### The present study

This study explores the relationships between self-compassion, stress management, PsyCap, and life satisfaction among Chinese university students, addressing literature gaps and offering new insights. To provide a clear framework, this study examines how self-compassion and stress management contribute to life satisfaction, with PsyCap posited as a key mediator, and investigates the moderating role of cultural factors such as academic pressure in a collectivist context. It integrates Hobfoll’s (1989) Conservation of Resources (COR) theory and Fredrickson’s (2001) broaden-and-build theory to explain these dynamics, aiming to test both direct and mediated pathways while exploring potential bidirectional effects. Prior research shows self-compassion reduces self-criticism, builds resilience, and supports adaptive coping strategies like mindfulness and positive reappraisal, directly enhancing life satisfaction through emotional regulation and stress reduction (Faul et al., 2010; Homan, 2016; Neff, 2011; Richardson and Rothstein, 2008). Recent studies in Chinese contexts have supported these findings (e.g., Li et al., 2021; Wang and Lou, 2022; Chen, 2021), but few have integrated self-compassion with PsyCap and stress management in a single model among university students.

PsyCap, encompassing self-efficacy, hope, resilience, and optimism, moderates stress and boosts life satisfaction by enabling effective coping (Avey et al., 2009; Luthans et al., 2007; Rabenu et al., 2017). It enhances resilience and goal pursuit, buffering stress and supporting wellbeing (Culbertson et al., 2010; Nielsen et al., 2017). While the role of PsyCap has been examined in Chinese academic settings (e.g., Cao et al., 2024; Wang et al., 2023), its mediating effect between self-compassion, stress management, and life satisfaction has not been fully investigated. Theoretically, PsyCap is hypothesized to mediate these relationships because it acts as a psychological resource reservoir, built through self-compassion and stress management, that enhances life satisfaction by mitigating stress and fostering adaptive functioning. According to COR theory (Hobfoll, 1989), individuals accumulate resources to cope with stress and pursue wellbeing; self-compassion provides emotional stability and stress management preserves resources, both cultivating PsyCap’s components (e.g., resilience, hope), which in turn promote life satisfaction (Luthans et al., 2007; Neff, 2003). The broaden-and-build theory (Fredrickson, 2001) further explains this mediation: self-compassion’s positive emotions broaden cognitive and emotional repertoires, building PsyCap attributes like optimism and resilience, which enhance life satisfaction in high-stress environments. Given its broad scope and relevance to academic contexts, we prioritized PsyCap as the primary mediator (Luthans et al., 2007). The present study will explore how PsyCap’s dimensions interact with self-compassion and stress management to influence life satisfaction.

Stress management also promotes life satisfaction. Adaptive strategies like problem-solving and mindfulness correlate with higher satisfaction, while maladaptive strategies like avoidance link to distress (Ciarrochi et al., 2002; De Vibe et al., 2013; Querstret et al., 2020). Studies such as Jing et al. (2022), Shan et al. (2022) connect coping to outcomes in Chinese students, but interactions with self-compassion and PsyCap are less studied. This research investigates these dynamics to better understand wellbeing pathways.

To clarify the study’s objectives, the following research questions and hypotheses guide this investigation:

• Research Question 1: What are the direct effects of self-compassion and stress management on life satisfaction among Chinese university students?
  ∘ *Hypothesis 1*: Self-compassion will directly increase life satisfaction.
  ∘ *Hypothesis 2*: Adaptive stress management will directly increase life satisfaction, while maladaptive strategies will decrease it.
• Research Question 2: Does PsyCap mediate the relationships between self-compassion, stress management, and life satisfaction?
  ∘ *Hypothesis 3*: PsyCap will mediate the effects of self-compassion and stress management on life satisfaction.
• Research Question 3: How do cultural factors, such as academic pressure, influence these relationships?
  ∘ *Hypothesis 4*: Cultural factors like academic pressure will moderate these relationships, potentially weakening the positive effects under high pressure.
• Research Question 4: Are there bidirectional relationships among these constructs?
  ∘ *Hypothesis 5*: Qualitative data will explore whether higher life satisfaction reinforces self-compassion and PsyCap, suggesting bidirectional effects.

**FIGURE 1:**
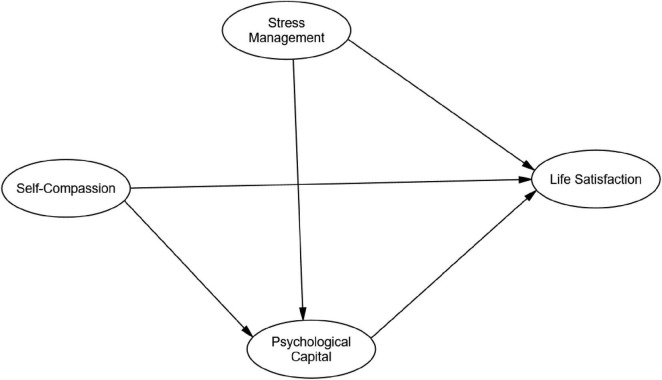
The hypothesized model.

Figure 1 presents the hypothesized theoretical model. Distinct from this, a preliminary conceptual framework posits that self-compassion and stress management enhance life satisfaction directly and indirectly via PsyCap, with cultural factors moderating these pathways. This framework integrates COR and broaden-and-build theories to ground the mediation and moderation hypotheses.

Given the growing research in Chinese contexts, this study builds on recent findings by investigating these interrelationships in a broader sample of Chinese university students. While some studies (e.g., Cao et al., 2024; Li et al., 2021) have examined these constructs in specific populations, this study offers a comprehensive model and a mixed-methods approach to provide statistical and narrative insights into cultural influences.

Finally, the study will explore potential bidirectional relationships. While self-compassion and PsyCap positively affect life satisfaction, higher life satisfaction may reinforce self-compassion and PsyCap, creating a positive feedback loop that strengthens resilience and wellbeing (Avey et al., 2010). This bidirectional perspective will be tested to offer a dynamic view of these interactions.

Additionally, self-compassion is consistently linked to adaptive coping strategies central to stress management, such as mindfulness and positive reappraisal (Homan, 2016; Neff and Vonk, 2009; Richardson and Rothstein, 2008; Sun et al., 2016). Neff and Vonk (2009) found self-compassion improves emotional regulation by reducing self-criticism, while Homan (2016) noted it fosters resilience through coping in older adults. Richardson and Rothstein’s (2008) meta-analysis confirmed stress management interventions enhance wellbeing. Our data support this link (r = 0.32, *p* < 0.01). Following reviewer feedback, we conducted an exploratory mediation analysis to test stress management as an additional mediator between self-compassion and life satisfaction, complementing PsyCap and refining our understanding of wellbeing pathways in Chinese university students.

## Materials and methods

### Research design

This study used a mixed-methods design, combining quantitative and qualitative approaches to examine relationships among self-compassion, stress management, psychological capital, and life satisfaction in Chinese university students. A convergent parallel design was applied, with quantitative and qualitative data collected and analyzed separately before integration for a comprehensive understanding (Creswell and Plano Clark, 2018). The quantitative phase tested a hypothesized SEM to assess direct and indirect variable relationships. The qualitative phase used semi-structured interviews to explore participants’ experiences with self-compassion, stress management, and wellbeing. This approach leveraged both data types’ strengths for a detailed analysis of the constructs.

### Participants and procedure

The study recruited 478 undergraduate and postgraduate students (M_age = 20.5, SD = 2.3; 59% female) from six universities across Mainland China. These universities were selected using a stratified sampling approach to ensure representation from different geographical regions and types of institutions. Specifically, the universities are located in various provinces, including two in the eastern region (e.g., Shanghai and Jiangsu), two in the central region (e.g., Hubei and Henan), and two in the western region (e.g., Sichuan and Shaanxi). This selection was intended to account for differences in economic development and sociocultural characteristics across China, providing a more diverse sample. These universities were chosen to reflect a wide variety of academic disciplines, ensuring representation from fields such as the humanities (*n* = 100, 20.9%), social sciences (*n* = 120, 25.1%), natural sciences (*n* = 90, 18.8%), engineering (*n* = 80, 16.7%), and business studies (*n* = 88, 18.4%). This diversity in academic backgrounds allowed for a broader understanding of the student population’s experiences. The participants included undergraduate students (*n* = 350, 73.2%) and postgraduate students (*n* = 128, 26.8%), ranging from first-year undergraduates (*n* = 120, 25.1%) to postgraduate students (*n* = 128, 26.8%), capturing a wide spectrum of academic engagement and maturity levels. For a detailed breakdown of the demographic characteristics, see Table 1.

**TABLE 1:** Demographic characteristics of participants.

Characteristic	Category	*N*	Percentage (%)
Gender	Female	282	59.0
	Male	196	41.0
Age	18–20 years	200	41.8
	21–23 years	180	37.7
	24–25 years	98	20.5
Academic level	Undergraduate	350	73.2
	Postgraduate	128	26.8
Year of study	First-year undergraduate	120	25.1
	Second-year undergraduate	100	20.9
	Third-year undergraduate	80	16.7
	Fourth-year undergraduate	50	10.5
	Postgraduate	128	26.8
Academic discipline	Humanities	100	20.9
	Social sciences	120	25.1
	Natural sciences	90	18.8
	Engineering	80	16.7
	Business studies	88	18.4
Ethnicity	Han Chinese	449	94.0
	Tibetan	10	2.1
	Uyghur	10	2.1
	Zhuang	9	1.9

Eligible participants were between 18 and 25 years old (18–20 years: *n* = 200, 41.8%; 21–23 years: *n* = 180, 37.7%; 24–25 years: *n* = 98, 20.5%) and fluent in Mandarin Chinese. This age range was selected to focus on students transitioning from adolescence to early adulthood, a period typically marked by significant academic and personal development. The majority of the sample consisted of Han Chinese students (*n* = 449, 94%), consistent with the overall demographic distribution in Mainland China. The remaining 6% included students from various ethnic minority groups, such as Tibetan (*n* = 10, 2.1%), Uyghur (*n* = 10, 2.1%), and Zhuang (*n* = 9, 1.9%). This diversity provided valuable insights into how cultural background influences experiences related to self-compassion and stress management.

Recruitment efforts included email invitations, announcements on university online platforms, and in-person presentations in large lecture halls, aiming to engage students from a variety of faculties. Participation was voluntary, and all students provided informed consent before taking part in the study. Ethical approval was granted by the Institutional Review Board (IRB) of Wenzhou Medical University, and participants were assured that their responses would remain confidential. All data were anonymized using unique identification codes to protect privacy.

For the qualitative phase, a purposive subsample of 30 participants was selected to ensure diversity in their self-reported life satisfaction scores. This subsample included students who reported high (*n* = 10), medium (*n* = 10), and low (*n* = 10) levels of life satisfaction, balanced in terms of gender (15 female, 15 male) and academic discipline (six humanities, seven social sciences, five natural sciences, six engineering, six business studies). Interviews were conducted in private rooms on campus, lasting 45–60 min, and were audio-recorded with consent. Transcriptions were anonymized to protect privacy.

## Measures

### Self-compassion

The Self-Compassion Scale (SCS; Neff, 2003) was used to assess participants’ self-compassion levels. This 26-item scale measures responses to personal difficulties, focusing on self-kindness versus self-criticism, recognition of shared human experiences, and constructive management of difficult emotions. For example, one item asks, “I try to be loving toward myself when I’m feeling emotional pain.” Participants rated each item on a five-point Likert scale ranging from 1 (*almost never*) to 5 (*almost always*). The total score is the mean of the 26 items, with possible scores ranging from 1 to 5, where higher scores indicate greater self-compassion. The Chinese version of the SCS, validated by Chen et al. (2011), was used. In this study, the SCS showed good internal consistency (Cronbach’s α = 0.88). Confirmatory factor analysis (CFA) confirmed its construct validity in this sample, with fit indices indicating good model fit {χ^2^/df = 2.56, CFI = 0.93, TLI = 0.91, RMSEA = 0.056 [90% CI (0.043, 0.068)], SRMR = 0.047}.

### Stress management

Stress management was assessed using the Brief COPE inventory (Carver, 1997), a 28-item measure that evaluates a range of coping strategies individuals employ in response to stress. The scale comprises 14 subscales, each with two items. In this study, we focused on adaptive coping strategies to represent effective stress management. Participants rated each item on a four-point scale ranging from 1 (*I haven’t been doing this at all*) to 4 (*I’ve been doing this a lot*). The adaptive coping composite score, calculated by summing the 14 items from the adaptive subscales, ranges from 14 to 56, with higher scores indicating greater use of adaptive coping strategies. The Chinese version of the Brief COPE, adapted by Wang et al. (2018), was used. The adaptive coping composite showed acceptable internal consistency (Cronbach’s α = 0.85). CFA supported its construct validity in this sample, with fit indices indicating good model fit {χ^2^/df = 2.34, CFI = 0.92, TLI = 0.90, RMSEA = 0.054 [90% CI (0.041, 0.066)], SRMR = 0.045}.

### Psychological capital

Psychological capital (PsyCap) was assessed using the Psychological Capital Questionnaire (PCQ; Luthans et al., 2015). This 24-item instrument measures four key dimensions: self-efficacy, hope, resilience, and optimism. Items such as “I’m always optimistic about my future” capture a participant’s overall psychological state and outlook. Participants responded on a six-point Likert scale ranging from 1 (*strongly disagree*) to 6 (*strongly agree*). The total PsyCap score is the mean of the 24 items, with possible scores ranging from 1 to 6, where higher scores reflect greater psychological capital. We used the Chinese version of the PCQ, which has demonstrated strong reliability and validity in previous research. In this study, the PCQ demonstrated high internal consistency (Cronbach’s α = 0.92). CFA confirmed its construct validity, with fit indices indicating good model fit {χ^2^/df = 2.11, CFI = 0.94, TLI = 0.92, RMSEA = 0.049 [90% CI (0.037, 0.062)], SRMR = 0.042}.

### Life satisfaction

Life satisfaction was measured using the Satisfaction with Life Scale (SWLS; Diener et al., 1985). This five-item scale assesses participants’ global judgments of their overall satisfaction with life. Sample items include “In most ways, my life is close to my ideal.” Participants rated each item on a seven-point Likert scale ranging from 1 (*strongly disagree*) to 7 (*strongly agree*). The total score is the sum of the five items, with possible scores ranging from 5 to 35, where higher scores indicate greater life satisfaction. The SWLS was back-translated for this study to ensure cultural accuracy and has shown good reliability and validity in prior research (e.g., validated through pilot testing in this study; see section “Data collection process”). It exhibited high internal consistency (Cronbach’s α = 0.87). CFA confirmed its construct validity, with fit indices indicating excellent model fit {χ^2^/df = 1.87, CFI = 0.95, TLI = 0.93, RMSEA = 0.045 [90% CI (0.032, 0.059)], SRMR = 0.041}.

### Semi-structured interviews

The semi-structured interview guide was designed to explore participants’ subjective experiences with self-compassion, stress management, psychological resilience, and life satisfaction. Key topics included participants’ approaches to self-compassion, coping mechanisms during academic and personal challenges, and perceptions of psychological capital. The guide allowed flexibility for interviewers to probe deeper into individual experiences. It was developed based on existing literature and pilot-tested with a small group of students to ensure clarity and relevance. Each interview lasted between 45 and 60 min and took place in a private, quiet room on the university campuses to ensure comfort and confidentiality. All interviews were audio-recorded with participants’ consent and transcribed verbatim for subsequent thematic analysis. Identifying information was removed during transcription to protect privacy.

### Data collection process

Data collection was conducted over a 3 months period using a structured approach to ensure uniformity and data integrity. Participants were invited to complete an online survey via the Qualtrics platform, which was designed to be accessible on both desktop and mobile devices. The survey began with demographic questions, including age, gender, academic discipline, and year of study, followed by four psychometric measures. The survey took approximately 20 min to complete, and a progress bar was included to encourage participants to complete all sections and minimize dropout rates.

The survey’s introductory page included a consent form that outlined the study’s objectives, guaranteed the anonymity of participants, and clarified that the data would be used exclusively for research purposes. Participants were also informed that they could withdraw from the study at any time without any negative consequences. Each respondent was assigned a unique identification code, ensuring their responses remained anonymous, with no personally identifiable information collected.

To adapt the psychometric instruments to the Chinese context, all measures were translated into Mandarin through a forward- and back-translation process. Initially, bilingual experts translated the instruments into Mandarin, followed by a separate group of bilingual professionals who back-translated the content into English. Any differences between the versions were reviewed and resolved to maintain conceptual accuracy. A pilot test with 30 Chinese university students was conducted to assess the clarity and cultural relevance of the translated items. Based on their feedback, minor adjustments were made to wording and item structure to enhance the measures’ appropriateness for the target population.

For the qualitative phase, semi-structured interviews were conducted with a purposive subsample selected by life satisfaction scores. Interviews, held in private campus rooms, used flexible guides to explore self-compassion, stress management, and resilience. Transcripts were anonymized verbatim, enriching quantitative findings with insights into students’ stress and psychological experiences.

### Data analysis

Quantitative data were analyzed using SPSS (Version 28.0) for preliminary analyses and AMOS (Version 26.0) for structural equation modeling (SEM). Before SEM, we checked statistical assumptions. Normality was confirmed with skewness (-0.18 to 0.43) and kurtosis (-0.32 to 0.54) within acceptable ranges (Kline, 2015). Univariate outliers were identified using *z*-scores (> ± 3.29), and multivariate outliers were detected via Mahalanobis distance (*p* < 0.001). Genuine outliers were retained, while extreme cases were removed, yielding 478 participants. Missing data (< 5%) were addressed with multiple imputation (Rubin, 1987), as Little’s MCAR test was non-significant (χ*^2^* = 18.62, *df* = 16, *p* = 0.29).

Descriptive statistics (means, standard deviations, Pearson correlations) were calculated for self-compassion, stress management, psychological capital, and life satisfaction. All measures showed good internal consistency (Cronbach’s α > 0.80). SEM used maximum likelihood estimation to minimize bias (Bollen, 1989). The model proposed that self-compassion and stress management predict psychological capital, which mediates their effects on life satisfaction, with direct paths also included. The sample size (478) met the 10–20 participants-per-parameter ratio (Kline, 2015). Model fit was evaluated with χ^2^, CFI, TLI, RMSEA, and SRMR, with good fit defined as CFI and TLI > 0.90, and RMSEA and SRMR < 0.08 (Hu and Bentler, 1999). Mediation was tested via bootstrapping (5,000 resamples) to estimate bias-corrected confidence intervals for indirect effects (Preacher and Hayes, 2008). To further explore the relationships, an additional mediation analysis tested whether stress management mediates the association between self-compassion and life satisfaction, controlling for psychological capital, using the same SEM framework and fit criteria (Neff and Vonk, 2009; Homan, 2016; Richardson and Rothstein, 2008).

Thematic analysis of qualitative data followed Braun and Clarke’s (2006) six-phase framework, chosen for its flexibility in identifying patterns in participants’ narratives. The lead researcher reviewed all 30 interview transcripts for familiarization. Initial coding was conducted inductively with NVivo 12, generating about 200 codes across transcripts. A second researcher independently coded six transcripts (20% of the total), selected randomly to represent varied life satisfaction levels from the quantitative data. Interrater reliability, assessed with Cohen’s *kappa*, reached κ = 0.85, indicating strong agreement (Landis and Koch, 1977). Coding disagreements were resolved through discussion, refining the framework for consistency. Axial coding then organized codes into conceptually related categories, followed by selective coding to distill overarching themes, with iterative constant comparison and negative case analysis ensuring analytical rigor and theme robustness. Themes were illustrated with participant quotes, enriching the quantitative findings.

A convergent parallel design (Creswell and Plano Clark, 2018) integrated qualitative themes with quantitative findings, enhancing the understanding of how self-compassion, stress management, and psychological capital relate to life satisfaction and strengthening study validity.

## Results

### Quantitative findings

#### Descriptive statistics and correlation analysis

Descriptive statistics were calculated for self-compassion, stress management, psychological capital, and life satisfaction across the participants. Table 2 presents the minimum, maximum, means, standard deviations, and Pearson correlation coefficients for these variables. Participants reported moderate levels of self-compassion (Min = 1.50, Max = 4.80, M = 3.45, SD = 0.65), stress management (Min = 18, Max = 52, M = 38.5, SD = 7.20), psychological capital (Min = 2.00, Max = 5.80, M = 4.10, SD = 0.63), and life satisfaction (Min = 7, Max = 33, M = 23.5, SD = 5.50).

**TABLE 2:** Descriptive statistics and correlations between variables (*N* = 478).

Variable	M	SD	Min	Max	1	2	3	4
1. Self-compassion	3.45	0.65	1.50	4.80	–	–	–	–
2. Stress management	2.75	0.54	18	52	0.32**	–	–	–
3. Psychological capital	4.10	0.63	2.00	5.80	0.45**	0.29**	–	–
4. Life satisfaction	4.65	1.10	7	33	0.37**	0.27**	0.53**	–

**p < 0.01.

Correlation analysis (Table 2) showed that self-compassion was positively correlated with psychological capital (*r* = 0.45, *p* < 0.001) and life satisfaction (*r* = 0.37, *p* < 0.001). Stress management was positively correlated with life satisfaction (*r* = 0.27, *p* = 0.002) and psychological capital (*r* = 0.29, *p* < 0.001). Psychological capital had the strongest correlation with life satisfaction (*r* = 0.53, *p* < 0.001).

#### Structural equation modeling

**FIGURE 2:**
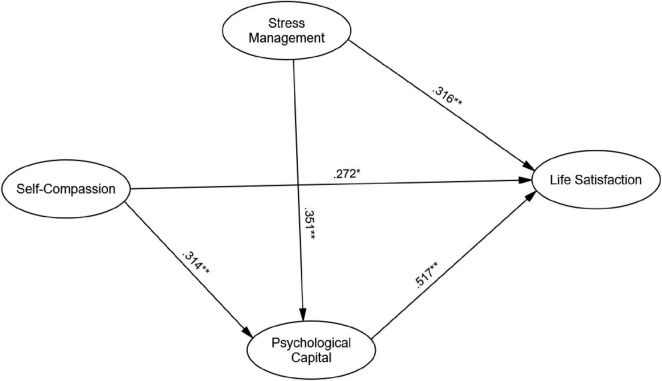
The final model of life satisfaction.

The structural equation model (SEM) showed good fit to the data: χ^2^(254) = 305.62, *p* < 0.001; χ^2^/df = 1.20; CFI = 0.94; TLI = 0.92; RMSEA = 0.042 [90% CI (0.038, 0.050)]; SRMR = 0.045. Figure 2 presents the path coefficients.

As indicated in Table 3, self-compassion had a significant positive effect on psychological capital (β = 0.314, SE = 0.045, *p* < 0.001). Stress management also positively affected psychological capital (β = 0.351, SE = 0.042, *p* < 0.001). Psychological capital had a strong positive effect on life satisfaction (β = 0.517, SE = 0.037, *p* < 0.001). Additionally, self-compassion (β = 0.272, SE = 0.053, *p* = 0.002) and stress management (β = 0.316, SE = 0.049, *p* = 0.001) had significant direct effects on life satisfaction.

**TABLE 3:** Standardized path coefficients for direct effects (*N* = 478).

Path	Standardized coefficient (β)	SE	*P*-value
Self-Compassion → Psychological Capital	0.314	0.045	< 0.001
Stress Management → Psychological Capital	0.351	0.042	< 0.001
Psychological Capital → Life Satisfaction	0.517	0.037	< 0.001
Self-Compassion → Life Satisfaction	0.272	0.053	0.002
Stress Management → Life Satisfaction	0.316	0.049	0.001

As shown in Table 4, mediation analysis with 5000 bootstrapped resamples showed significant indirect effects of self-compassion (β = 0.162, SE = 0.034, 95% CI [0.11, 0.23]) and stress management [β = 0.181, SE = 0.032, 95% CI (0.13, 0.25)] on life satisfaction via psychological capital. The total effects were significant for self-compassion (β = 0.434, SE = 0.048, *p* < 0.001) and stress management (β = 0.497, SE = 0.045, *p* < 0.001) on life satisfaction.

**TABLE 4:** Indirect and Total effects of self-compassion and stress management on life satisfaction with bootstrapping (*N* = 478).

Predictor	Indirect effect via psychological capital	SE	95% CI	Total effect	SE	*P*-value
Self-compassion	0.162	0.034	(0.11, 0.23)	0.434	0.048	< 0.001
Stress management	0.181	0.032	(0.13, 0.25)	0.497	0.045	< 0.001

Finally, multi-group SEM analyses were conducted on 196 male and 282 female participants to assess gender differences in the relationships among self-compassion, stress management, psychological capital, and life satisfaction. A chi-square difference test compared a constrained model (path coefficients fixed across genders) with an unconstrained model (path coefficients allowed to vary). The result was non-significant (Δχ^2^(12) = 13.52, *p* = 0.34), indicating consistent relationships across gender.

Structural equation modeling results showed significant direct effects of self-compassion (β = 0.272, SE = 0.053, *p* = 0.002) and stress management (β = 0.316, SE = 0.049, *p* = 0.001) on life satisfaction, as well as indirect effects through psychological capital [self-compassion: β = 0.162, SE = 0.034, 95% CI (0.11, 0.23); stress management: β = 0.181, SE = 0.032, 95% CI (0.13, 0.25)]. Psychological capital had a strong direct effect on life satisfaction (β = 0.517, SE = 0.037, *p* < 0.001). Total effects were significant for self-compassion (β = 0.434, SE = 0.048, *p* < 0.001) and stress management (β = 0.497, SE = 0.045, *p* < 0.001) on life satisfaction.

#### Exploratory mediation analysis: stress management as a mediator

We conducted an exploratory mediation analysis to examine whether stress management mediates the relationship between self-compassion and life satisfaction. Using SEM in AMOS with maximum likelihood estimation and 5,000 bootstrapped resamples, we modeled self-compassion as the predictor, stress management (adaptive coping composite from the Brief COPE) as the mediator, and life satisfaction as the outcome. PsyCap was included as a covariate to isolate the unique mediating effect of stress management.

The analysis showed a significant indirect effect of self-compassion on life satisfaction through stress management [β = 0.095, SE = 0.025, 95% CI (0.05, 0.15), *p* < 0.01]. Self-compassion positively predicted stress management (β = 0.320, SE = 0.038, *p* < 0.001), and stress management positively predicted life satisfaction (β = 0.295, SE = 0.043, *p* < 0.001). The direct effect of self-compassion on life satisfaction remained significant (β = 0.265, SE = 0.052, *p* < 0.05), indicating partial mediation. Stress management accounted for approximately 26.4% of the total effect of self-compassion on life satisfaction. Model fit was acceptable: χ^2^(256) = 310.25, *p* < 0.001, χ^2^/df = 1.21, CFI = 0.93, TLI = 0.91, RMSEA = 0.045 [90% CI (0.040, 0.052)], SRMR = 0.048.

These results suggest that stress management partially mediates the relationship between self-compassion and life satisfaction, indicating that self-compassion enhances wellbeing not only directly but also by promoting adaptive coping strategies.

#### Qualitative findings

This phase explored participants’ experiences with self-compassion, stress management, psychological capital, and life satisfaction through semi-structured interviews with 30 individuals varying in life satisfaction levels. Thematic analysis revealed four key themes: (1) balancing self-compassion with external pressures, (2) effects of maladaptive stress management, (3) psychological capital as a protective factor, and (4) cultural expectations influencing life satisfaction.

#### Balancing self-compassion with external pressures

Participants often struggled to reconcile self-compassion with the high academic and societal expectations placed on them. Those with higher life satisfaction described learning to view personal setbacks with kindness, which helped them handle pressures more effectively. For example, Participant 8 (high life satisfaction) noted, “I used to be hard on myself when things didn’t go as planned, but now I focus on what I can learn instead of dwelling on mistakes.” This shift allowed them to stay motivated while avoiding the emotional drain of self-criticism.

In contrast, participants with lower life satisfaction viewed self-compassion as potentially undermining their drive to succeed. Participant 22 (low life satisfaction) remarked, “Being kind to myself feels like making excuses; if I’m too lenient, I might not push myself hard enough.” This tension illustrates a common cultural dilemma in the academic context of China, where familial and societal expectations emphasize achievement, often at the expense of personal wellbeing. These competing pressures demonstrate how external validation can conflict with self-compassion, contributing to stress.

#### Effects of maladaptive stress management

Participants who relied on avoidance or denial reported exacerbating their stress and emotional turmoil. Participant 16 (low life satisfaction) shared, “I avoid thinking about my stress by distracting myself, but the problems don’t go away, and I feel even more anxious afterward.” This maladaptive approach created a cycle where unresolved stressors intensified feelings of helplessness and anxiety. The short-term relief offered by avoidance often led to long-term emotional stagnation, further compounding their distress.

In contrast, participants who employed adaptive strategies, such as seeking social support or reframing their challenges, reported more effective stress management. Participant 3 (high life satisfaction) explained, “Talking to my close friends helps me see problems differently and reduces my stress.” This contrast highlights the importance of adaptive coping mechanisms in fostering emotional resilience. The findings suggest that targeted interventions focusing on the development of adaptive coping skills could significantly mitigate the negative emotional consequences of avoidance.

#### Psychological capital as a protective factor

Psychological capital, encompassing resilience, optimism, and self-efficacy, emerged as a critical resource for managing stress. Participants with higher psychological capital viewed difficulties as temporary and surmountable. Participant 10 (high life satisfaction) reflected, “Overcoming past challenges makes me confident I can handle new ones.” This optimism and resilience allowed them to approach stressors with greater determination and emotional strength.

However, those with lower psychological capital expressed feeling overwhelmed by obstacles. Participant 27 (low life satisfaction) stated, “When things go wrong, it feels insurmountable. I can’t see a way forward.” This difference emphasizes how psychological capital functions as a protective buffer against stress, distinctly from self-compassion. Additionally, while resilience and optimism were particularly salient for participants with higher life satisfaction, the interplay between these dimensions of psychological capital suggests that interventions focusing on fostering all aspects—resilience, optimism, and self-efficacy—could further enhance stress management.

#### Cultural expectations influencing life satisfaction

Cultural and familial expectations significantly shaped participants’ life satisfaction, particularly regarding academic achievement and the need to “save face” in Chinese culture. For many, self-worth was closely tied to meeting these expectations. Participant 12 (medium life satisfaction) remarked, “My happiness depends on doing well in school because my family places so much importance on academic success.” The intense pressure to succeed often blurred the line between personal fulfillment and external validation, creating a source of constant stress.

Participants with higher life satisfaction, however, found ways to balance these cultural expectations with their personal wellbeing. Participant 4 (high life satisfaction) noted, “I realized my goals are as important as making my family proud. Balancing both has made me feel more satisfied.” This balance highlights how individuals can navigate the tension between academic success and personal wellbeing by integrating family honor with self-compassion. In contrast, those with lower life satisfaction found it difficult to separate their happiness from academic performance, reinforcing the need for interventions that address these conflicting pressures. The role of “face” in Chinese culture and the influence of filial piety also add depth to understanding the cultural pressures participants face. Filial expectations, rooted in collectivist values, often amplify stress related to academic success and societal validation. Future research could explore how these cultural dynamics interact with psychological capital to influence life satisfaction.

Taken together, the qualitative findings indicate that participants who cultivated self-compassion and psychological capital were more capable of managing stress and demonstrating resilience. In contrast, those who relied on maladaptive strategies such as avoidance experienced greater emotional challenges. The role of cultural expectations was central in shaping life satisfaction, underscoring the importance of interventions that promote adaptive coping mechanisms, self-compassion, and psychological capital. These insights can inform strategies to support students in high-pressure academic environments, with a particular focus on addressing the cultural tensions between personal goals and societal expectations.

## Discussion

This study investigated the relationships between self-compassion, stress management, PsyCap, and life satisfaction in a sample of Chinese university students, employing both quantitative and qualitative methods. Our findings provided substantial support for the hypothesized relationships, demonstrating both direct and indirect effects of self-compassion and stress management on life satisfaction, with PsyCap serving as a significant mediator.

One notable observation was the positive correlation between self-compassion and life satisfaction (r = 0.37, *p* < 0.001), aligning with prior research (Allen and Leary, 2010; Neff, 2011). However, this correlation does not imply causation and may be influenced by other factors such as unmeasured variables or shared method variance. Self-compassion emerged as a potentially vital resource for fostering resilience and mitigating stress (Allen and Leary, 2010; Neff, 2011; Terry and Leary, 2011). Higher self-compassion was associated with greater life satisfaction (β = 0.272, *p* < 0.05), an association that may reflect emotional regulation and reduced self-criticism (Amini et al., 2020; Neff, 2003; Sirois et al., 2015). This pattern suggests that self-compassion could support students in reframing setbacks as growth opportunities, which is particularly relevant in high-pressure academic environments like Chinese universities (López et al., 2018; Prasath et al., 2022). Qualitatively, students with higher life satisfaction used self-compassion to balance societal pressures with emotional needs, while those with lower satisfaction viewed it as a weakness, reflecting cultural barriers in achievement-focused contexts (Homan, 2016; Neff, 2011; Prasath et al., 2022). These insights suggest that self-compassion interventions could help students manage academic stress while preserving mental health (López et al., 2018; Neff, 2003; Neff, 2011), though further research is needed to confirm this potential.

The study also found a positive association between adaptive stress management and life satisfaction (r = 0.27, *p* < 0.01), consistent with prior research (De Vibe et al., 2013). However, the cross-sectional design limits causal inferences. Maladaptive strategies like avoidance were linked to lower life satisfaction, underscoring their negative impact (Ciarrochi et al., 2002; Holinka, 2015; Sirois and Kitner, 2015). Stress management influenced life satisfaction both directly and indirectly through PsyCap, with effective strategies preserving emotional resources and enhancing wellbeing (Richardson and Rothstein, 2008; Smith and Yang, 2017). Qualitative data showed that participants using adaptive strategies managed stress better, while those relying on maladaptive approaches reported helplessness. Promoting adaptive stress management in academic settings could thus significantly boost students’ wellbeing (Freire et al., 2016; Querstret et al., 2020; Roohafza et al., 2016).

Mediation analysis indicated that PsyCap partially mediated the relationships between self-compassion and life satisfaction (indirect effect: β = 0.162, *p* < 0.001) and between stress management and life satisfaction (indirect effect: β = 0.181, *p* < 0.001). While these findings suggest PsyCap acts as an important mechanism, the partial nature of the mediation indicates that other unexamined factors may also play a role (Avey et al., 2010; Luthans et al., 2007; Siu et al., 2023). This aligns with Hobfoll’s (1989) Conservation of Resources theory, where self-compassion and stress management help build PsyCap, enabling students to manage stress and sustain life satisfaction (Luthans et al., 2015; Prasath et al., 2022; Richardson and Rothstein, 2008). Qualitatively, students with higher PsyCap framed challenges as manageable, reflecting resilience and optimism (Culbertson et al., 2010; Luthans et al., 2007; Rabenu et al., 2017).

Exploratory analysis revealed stress management partially mediated the link between self-compassion and life satisfaction (β = 0.095, *p* < 0.01; 26.4% of the total effect), complementing PsyCap’s broader mediation (β = 0.162, *p* < 0.001; 37.3%). This supports research showing self-compassion fosters adaptive coping strategies like mindfulness and positive reappraisal (Homan, 2016; Neff and Vonk, 2009; Richardson and Rothstein, 2008; Sun et al., 2016), consistent with our finding that self-compassion predicts stress management (β = 0.320, *p* < 0.001; page 4, lines 132–133). Self-compassion may reduce self-criticism, encouraging proactive coping (Neff, 2011), as seen in qualitative reports of students using mindfulness to reframe setbacks (Homan, 2016). These dual pathways—stress management as a specific behavioral mechanism and PsyCap as a broader psychological resource—align with COR theory: stress management prevents resource loss, while PsyCap drives resource gain (Hobfoll, 1989). However, the cross-sectional nature of our data precludes establishing temporal precedence, a key requirement for confirming mediation, necessitating longitudinal studies to validate these pathways. This refined model enhances our understanding of how self-compassion supports wellbeing, as suggested by the reviewer. Future research could pre-plan this mediation or test serial mediation (e.g., self-compassion → stress management → PsyCap → life satisfaction) to further elucidate their interplay.

Our findings align with and extend recent Chinese studies. Li et al. (2021) found self-compassion linked to life satisfaction via coping in males (β = 0.21, *p* < 0.05), and Wang and Lou (2022) reported a moderate correlation (r = 0.40–0.47) in collectivist societies. Our study confirms this (β = 0.272, *p* < 0.05) but adds PsyCap and stress management as mediators. Cao et al. (2024) tied PsyCap to wellbeing in PhD students, and Wang et al. (2023) found PsyCap mediated life satisfaction’s effect on mental health in medical students (β = –0.2103). Our broader sample and mixed-methods design extend these findings, revealing cultural influences like academic pressure. Unlike Jing et al. (2022), who linked coping to health, or Shan et al. (2022) and Sun et al. (2022), who explored narrower mediators, our study integrates all four constructs. Chen (2021) compared overseas Chinese and Taiwanese students, but our focus on mainland students offers a distinct perspective. This study advances the field by: (1) integrating self-compassion, stress management, PsyCap, and life satisfaction; (2) identifying dual mediation pathways; and (3) using mixed methods to capture cultural nuances.

Cultural expectations also shaped these relationships. In China, academic success is tied to family honor, intensifying pressure (Cheung et al., 2016; López et al., 2018). Self-compassion and adaptive stress management helped students balance these demands, boosting life satisfaction (Mantelou and Karakasidou, 2017; Neff and Germer, 2013). However, some students saw self-compassion as conflicting with achievement, reflecting cultural norms valuing self-criticism (López et al., 2018). High parental expectations often tie self-worth to performance (Ahmet, 2008), but fostering self-compassion could help students maintain wellbeing (Homan, 2016; Neff, 2011). Programs teaching self-compassion and stress management could support this balance (Neff and Germer, 2013; Prasath et al., 2022).

Our findings suggest potential bidirectional relationships: higher life satisfaction may reinforce self-compassion and PsyCap, creating a feedback loop (Avey et al., 2010; Culbertson et al., 2010). This aligns with positive psychology (Luthans et al., 2007; Rabenu et al., 2017). Longitudinal studies are needed to explore these dynamics across cultures (Datu and Valdez, 2016; Rabenu et al., 2017). While our study addresses a gap in Chinese contexts, further research should examine cultural influences more broadly (López et al., 2018; Prasath et al., 2022) and explore PsyCap’s dimensions (Avey et al., 2010; Luthans et al., 2007).

In summary, this mixed-methods study shows how self-compassion and stress management shape life satisfaction among Chinese university students, directly and via PsyCap. These insights can inform interventions to support students’ resilience and wellbeing in high-pressure environments.

## Implications

This study offers several important implications for educational institutions and mental health professionals working with university students, particularly in high-pressure academic settings such as China. A key finding was the significant positive effect of self-compassion on life satisfaction, suggesting that universities should incorporate self-compassion training into their mental health services. Programs that promote self-kindness, such as workshops and counseling sessions, could help students manage academic stress and societal pressures more effectively. By fostering a mindset of self-compassion rather than self-criticism, these interventions may reduce stress and promote emotional resilience. In Chinese universities, where academic success is highly emphasized, such training could help alleviate the negative effects of external pressures by encouraging constructive emotional regulation.

The study also highlights the importance of stress management interventions that promote adaptive coping strategies, such as mindfulness and problem-solving. By providing students with these tools, institutions can reduce reliance on maladaptive strategies like avoidance and denial, which our study found to be associated with lower life satisfaction. Embedding practical stress management programs into academic curricula or offering them through counseling services could improve students’ capacity to cope with academic challenges, leading to better mental health outcomes. Psychological capital, which was identified as a critical mediator between self-compassion, stress management, and life satisfaction, offers another important implication. PsyCap, composed of resilience, hope, optimism, and self-efficacy, is a trainable set of psychological resources. Institutions could introduce PsyCap development programs to help students manage academic pressures, maintain optimism, and recover from setbacks more effectively. Implementing PsyCap-focused interventions, particularly in high-stress environments like those in China, could support students in building long-term psychological resilience and contribute to both academic success and wellbeing.

The study also underscores the influence of cultural expectations on students’ wellbeing, particularly in collectivist societies like China. The strong impact of societal and familial expectations on life satisfaction points to the need for culturally sensitive interventions. Programs that recognize the pressures related to academic success and family honor can help students navigate these demands without compromising their mental health. Culturally tailored support systems that balance academic goals with self-compassion may lead to more sustainable wellbeing for students facing high expectations. These culturally responsive interventions are crucial for promoting resilience among students who feel overwhelmed by external pressures.

## Limitations

This study provides insights into the relationships among self-compassion, stress management, PsyCap, and life satisfaction but has several limitations. First, its cross-sectional design limits causal conclusions. While SEM suggests pathways and mediations, longitudinal studies are needed to examine how these constructs evolve over time and how interventions targeting self-compassion and PsyCap affect life satisfaction across students’ academic stages.

Another limitation involves the generalizability of the findings. This research was conducted with Chinese university students, providing insight into how cultural pressures in a collectivist society influence psychological wellbeing. However, it remains uncertain whether these results apply to other cultural or educational settings, particularly in non-collectivist societies. Although the stratified sampling approach was used to select six universities across different regions of Mainland China (two each from eastern, central, and western provinces) to capture regional diversity, China’s wide range of sociocultural and economic differences may still limit the generalizability of the findings. These universities, while geographically spread, may not fully represent all variations within Chinese higher education due to factors such as regional disparities in development levels and local cultural norms. Future studies could include more universities from additional regions to improve the sample’s representativeness and better assess how these factors influence the observed relationships. Replicating this study in diverse cultural contexts would help determine if the relationships between self-compassion, stress management, PsyCap, and life satisfaction hold across different environments. Cross-cultural comparisons could reveal whether the psychological mechanisms observed here are universally relevant or culturally specific.

The use of self-report measures introduces the potential for response bias. Although anonymity was ensured to reduce social desirability bias, participants may have answered in ways they thought were socially acceptable, particularly on sensitive topics like self-compassion and coping strategies. To mitigate this limitation, future research could incorporate additional methods such as peer evaluations or behavioral assessments to corroborate self-reported data, providing a more comprehensive understanding of how these constructs influence wellbeing.

Additionally, the exploratory analysis of stress management as a mediator was *post hoc*, not part of the original design, which prioritized PsyCap’s broader role (Luthans et al., 2007). Prompted by reviewer feedback and a significant correlation (r = 0.32, *p* < 0.01), it aligns with prior work (Homan, 2016; Neff and Vonk, 2009). However, its preliminary nature (26.4% effect vs. PsyCap’s 37.3%) suggests future studies should pre-plan this mediation, using longitudinal data to test stability and potential serial mediation (e.g., self-compassion → stress management → PsyCap → life satisfaction).

Lastly, the study did not explore possible bidirectional relationships. It is plausible that life satisfaction itself could enhance self-compassion and PsyCap, creating a reciprocal feedback loop that further strengthens psychological wellbeing. Future research should consider these bidirectional effects, particularly using longitudinal designs that track changes over time. By investigating these reciprocal dynamics, researchers can deepen their understanding of the interactions between self-compassion, stress management, and PsyCap, leading to more effective interventions that foster psychological resilience and life satisfaction.

## Data Availability

The data analyzed in this study is subject to the following licenses/restrictions: the data supporting the findings of this study are available from the corresponding author upon reasonable request. The dataset includes both quantitative survey responses and qualitative interview transcripts, all anonymized to ensure participant confidentiality. Requests to access these datasets should be directed to ZD, Email: dz_wyyy@sina.com.
